# Effects of an Aquatic Physical Exercise Program on Ventilatory Parameters in People with Parkinson's Disease

**DOI:** 10.1155/2022/2073068

**Published:** 2022-08-31

**Authors:** Bruna Yamaguchi, Dielise Debona Iucksch, Luis Henrique Paladini, Vera Lúcia Israel

**Affiliations:** Federal University of Paraná, Curitiba, Brazil

## Abstract

Problems in the respiratory system are the main cause of death in Parkinson's disease (PD). Ventilatory limitations can also be part of a vicious cycle involving physical-functional limitations (e.g., walking difficulties) and the patients' perception of fatigue. The objective of this study was to analyze the effects of an aquatic physical exercise intervention program on ventilatory parameters, perception of fatigue, and gait capacity in participants with PD. This quasi-experimental study had a single group with repeated measures in four assessments, proposing an aquatic physical exercise intervention program. The inclusion criteria encompassed being in levels 1 to 4 on the Hoehn and Yahr scale and having a medical certificate for the activities. Assessments took place at 3-month intervals between them—the first period was the control, the second following the intervention, and the third period was the follow-up. The intervention had 25 biweekly sessions over 3 months. A total of 13 people (71.3 ± 5.61 years old) participated in the intervention, without significant differences in the control period. Between the intervention assessments, they had statistically significant differences in MIP, MEP, FVC, Tiffeneau index, MVV, and fatigue. The study demonstrated that the aquatic physical exercise intervention was effective for ventilatory outcomes and fatigue in people with PD.

## 1. Introduction

Possible respiratory impairments in Parkinson's disease (PD) were described in 1817 by James Parkinson, who defined it as “paralysis agitans” [1, 2]. It has been currently reported that more people in this population die from pneumonia than same-age older adults [3] and that respiratory problem share the main cause of death in PD [1, 2]. Besides the mortality rates, respiratory limitations also impair PD patients' overall functioning, causing them to progressively lose their independence and quality of life [2–4].

Results in the literature present different causal approaches regarding the characteristics of pulmonary function in PD. Studies indicate sharper respiratory changes than in healthy older people [1–5], which seem to be also related to PD progression [2]. Furthermore, ventilatory limitations can be part of a vicious cycle involving physical-functional limitations (e.g., walking difficulties) and the PD patients' perception of fatigue [6, 7]. However, studies do not unanimously define whether there are obstructions, restrictions, mixed limitations, or even respiratory muscle weakness in people with PD. Hence, the literature still lacks clarifications regarding pulmonary function in PD.

Regarding multi-professional treatments of possible respiratory disorders in PD, physical therapy and other exercise-based strategies are characterized as clinically helpful activities [8]. Physical exercises can stimulate neurotrophic factors, with neuroprotection and neuroplasticity effects [9]. However, few studies approach interventions based on physical activity programs, whose main outcomes are the limitations related to ventilatory variables [10, 11]. The existing ones use incentive spirometry [12, 13], while few studies approach physical activities and/or ventilatory patterns [4, 14–16].

No studies were found approaching aquatic physical therapy to improve respiratory parameters associated with the perception of fatigue and gait capacity in PD. On the other hand, successful experiences with aquatic physical therapy intervention have been reported, with motor and quality-of-life outcomes for this population [17–22]. Aquatic exercises, whose movements are safely made in the pool, have likewise been experienced for other neurological [23]and motor neuron diseases [24], which impair ventilation [25, 26].

Besides the positive effects of aerobic exercises performed on land, the aquatic environment further favors ventilation because it potentially increases inspiratory force. It also triggers the rearrangement of blood circulation and volume in the chest, due to hydrostatic pressure when immersed, in combination with water temperature and other physical properties, and adequately prescribed aquatic physical activities [16–26]

Thus, the objective of the present study was to analyze the effects of an intervention program with aquatic physical activities on the ventilatory parameters, perception of fatigue, and gait capacity in participants with PD.

## 2. Methodology

This is a quasi-experimental single-group study [27] with repeated measures. The research complied with the guidelines in Resolution 466/12, of the National Health Council [28], and the Declaration of Helsinki, and was approved by the Research Ethics Committee.

**Figure 1:**
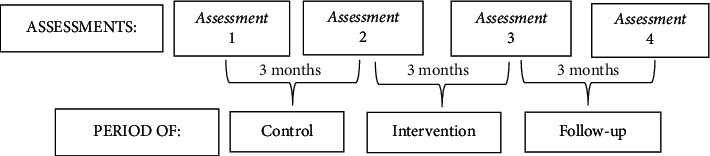
Outline of assessments and intervention.

The assessments took place in four moments. The 3 months between assessments 1 and 2 were the control period when participants continued their everyday activities. In the second period (between assessments 2 and 3), the group not only maintained their everyday activities but also joined the heated pool intervention program. There was no intervention in the period between the last two assessments (3 and 4), i.e., follow-up, when the group only maintained their everyday activities. The flowchart (Figure 1) shows the periods, the four assessment moments, and the time between the assessments.

Participants were recruited from an association of people with PD in a capital city in Southern Brazil. They were invited to the research and joined it by signing an informed consent form. The inclusion criteria were as follows: participants of both sexes, clinically diagnosed with idiopathic PD, in stages 1 to 4 on the Hoehn and Yahr scale, and with a medical certificate for performing physical activities and attending heated pools.

The exclusion criteria were as follows: not being able to walk independently of help from other people; having other diseases that might interfere with the physical assessments (e.g., balance changes of vestibular origin), visual or auditory sensory deficits that hindered them from following verbal and visual instructions, or any uncontrolled respiratory or cardiovascular diseases; having a history of pulmonary surgery, recent respiratory tract infection, or any absolute contraindication to attend heated pools; being absent in more than 10% of the intervention; changing physical activities or L-dopa-based drug intake parameters during the research period.

Participants were assessed with the Hoehn & Yahr scale and Montreal Cognitive Assessment (MoCA) and had their data collected (age, sex, time since PD diagnosis) for sample characterization.

**Figure 2:**
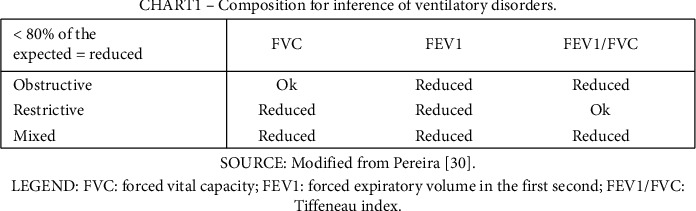
Composition for inference of ventilatory disorders.

As intervention-dependent outcomes, participants were assessed with a duly calibrated analogic respiratory pressure meter manufactured by Wika to measure their maximum inspiratory (MIP) and expiratory pressure (MEP). The assessment followed the instructions of the American Thoracic Society and European Respiratory Society [29]. The spirometry was made with a portable spirometer (brand and model: MIR/Spirobank G). The forced vital capacity (FVC), forced expiratory volume in the first second (FEV1), Tiffeneau index (i.e., the FEV1/FVC ratio), and maximum voluntary ventilation (MVV) were analyzed with criteria of the Brazilian Society of Pneumology [30]. Individual values below 80% of the expected per age, sex, height, and mass were inferred as at risk for obstructive, restrictive, or mixed respiratory disorders, as shown in Figure 2.

The Fatigue Severity Scale (FSS) was used for the perception of fatigue, with self-reported scores from 1 to 7—in which 1 indicated disagreement with the statement on fatigue, and 7 indicated strong agreement with it; higher values pointed to a greater perception of fatigue [31].

The 6-Minute Walk Test (6 MWT) was used to assess the physical capacity at submaximal effort [32].

### 2.1. Interventions

**Figure 3:**
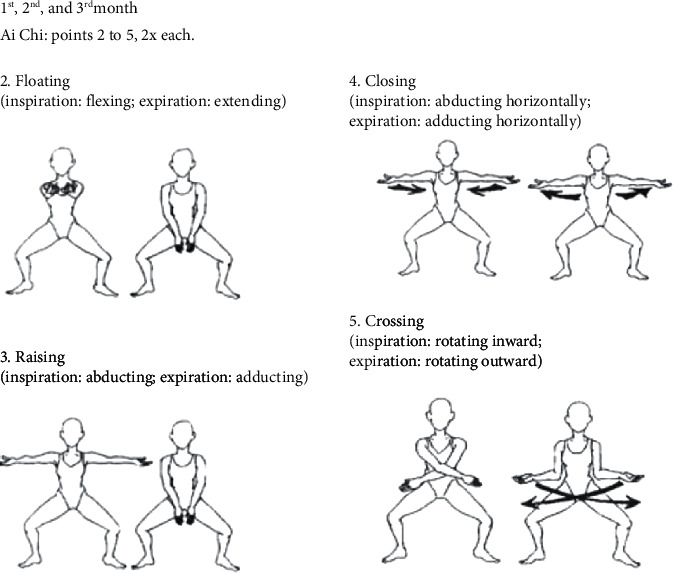
Phase of relaxation.

The aquatic environment interventions took place in small groups of participants in a 10.70 m long, 2.90 m wide, and 1.20 m deep pool, heated to approximately 33°C. There were 25 sessions over 3 months, held twice a week on non-consecutive days, lasting 50 minutes each. Every session had a warm-up, followed by specific exercises, and finished with a cooldown, following the recommendations of the European Physiotherapy Guideline for Parkinson's Disease [33]. The exercises approached the five aquatic motor learning phases, as proposed by Israel and Pardo (2000) [34], with emphasis on specialized therapeutic exercises and global organic conditioning. The aquatic intervention program is described in detail in Tables 1–4 and Figure 3.

The Borg 6–20 scale was used during exercise to control the reported exercise intensity, which was kept between 12 and 16 on the scale. This range enables physiological adaptations of the physical activity balanced with good tolerance to them [35–37].

### 2.2. Data Analysis Procedures

The measures of central tendency and dispersion and the normality of the sample distribution were verified. The mean values of the four assessments were compared with the repeated-measure ANOVA for different times. Mauchly's sphericity test was applied, and the Greenhouse-Geisser correction was used in the case of data whose sphericity was not assumed [38]. Afterward, the Bonferroni post hoc test was applied to the variables with statistical differences to verify between which assessments there were differences [38]. The statistical significance value was set at *p* < 0.05; the SPSS 22.0 program for Windows [39] was used.

## 3. Results

### 3.1. Sample Characterization

**Figure 4:**
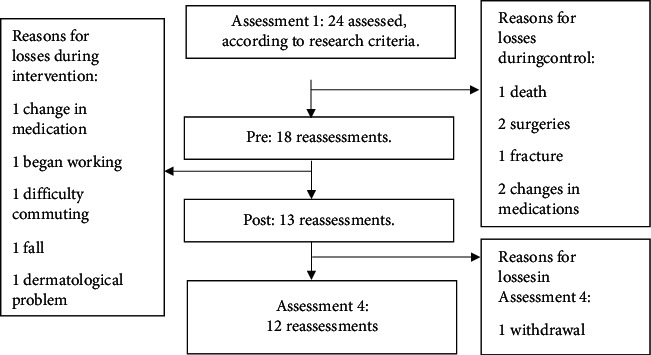
Flowchart of research sample losses.

Initially (Assessment1), 24 participants who met the inclusion and exclusion criteria were assessed. As shown in the flowchart, in Figure 4, there were sample losses, so only 12 subjects participated in the complete outcome analysis.

The characterization of subjects who finished the program is shown in Table 5.

### 3.2. Dependent Outcomes

#### 3.2.1. Ventilatory Variables

The muscle strength respiratory assessments and the spirometry flow and volume outcomes are given in mean, standard deviation, 95% confidence interval, and *p* value. When the difference was significant, the effect size and statistical power for these comparisons were calculated (Table 6).

The respiratory force variables had significant intervention-related differences. MIP increased significantly between Assessments 2 and 3 (*p*=0.026); however, comparing Assessments 3 and 4, the intervention gains did not remain after follow-up (*p*=0.024). There was also a statistical difference in MEP between Assessments 2 and 3 (*p* ≥ 0.001), demonstrating a post-intervention gain in expiratory force, which decreased afterward between Assessments 3 and 4 (*p*=0.009).

There were statistical differences in the spirometry means for FVC between Assessments 2 and 3 (*p*=0.015)—FVC increased after the intervention. MVV had statistical differences between Assessments 2 and 3 (*p* ≥ 0.001). MVV increased after the intervention and then significantly decreased between Assessments 3 and 4 (*p*=0.006).

Regarding classification with spirometry, there were two cases of restrictive and two of mixed ventilatory disorders in the sample in Assessments 1, 2, and 3. In Assessment 4, there were two cases of mixed and one restrictive ventilatory disorder, according to the described inference criteria.

The secondary outcomes, perception of fatigue and gait capacity, are described in Table 7. There were statistical differences in fatigue between Assessments 2 and 3 (*p* < 0.001), indicating a decrease in complaints of fatigue after the intervention program. However, the reported fatigue increased between Assessments 3 and 4 (*p*=0.006). Lastly, no difference was observed in the 6 MWT in the four research assessments.

## 4. Discussion

The proposed aquatic intervention had positive results in ventilatory variables and perception of fatigue, although it did not have a statistically significant increase in gait capacity assessed with 6 MWT. These results are relevant because respiratory impairments are greatly debilitating complications, expected in PD progression.

We determined the inclusion of participants with Hoehn and Yahr = 4, in order to include people with greater limitations in physical exercises, and we continue to recommend it for future studies. Few studies include severely ill patients, failing to propose activities that include this population. In addition, it seems that aquatic exercise is recommended more intensively for subjects with body balance disorders [40].

A review of aquatic exercise in PD included stages 1 to 4 of PD, showing no adverse effects in participants during aquatic therapy intervention. Our participants with Hoehn and Yahr = 4 also had no limitations in exercise participation and there were no issues related to unexpected submersion [41].

Respiratory muscle strength was one of the outcomes in this study that had positive results from the aquatic intervention in people with PD. On average, MIP increased by 18.25% and MEP, by 21.18% from pre- to post-intervention. Conducting the intervention in an aquatic environment possibly favored such gains because the water provides different ventilation stimuli from the land—the immersed chest suffers hydrostatic pressure, which creates inspiratory resistance and thus a type of overload [42]. The respiratory muscles responsible for inspiration need to surpass the overload, increasing respiratory strength [26]. Moreover, hydrostatic pressure is related to the depth and position of the body in the pool [26]. As the pool used for the intervention was 1.20 m deep, participants stayed with immersion between cervical (C2–C7) and upper thoracic (T1–T6) levels when in the standing posture. This favored respiratory training, especially inspiratory.

Upper trunk immersion in water also causes acute ventilation adaptations, as it increases inner pressure [26]. Blood circulation is redistributed, and its central volume increases due to vasoconstriction in the limbs [42], especially in those more deeply immersed in the pool [26].

The gain in expiratory strength performance in aquatic intervention, though seemingly not grounded on hydrostatic pressure, may depend on other factors more related to the physical activity, which likely stimulate muscle groups that aid forced expiration. Muscles such as the abdominal ones are known to promote forced expiratory strength [43]. Thus, as the water changes body control—often without a base of support [44, 45]—recruiting core and abdominal muscle control and strength, the aquatic setting stimulates the person to seek body stabilization, activating especially the trunk muscles.

Lower FVC spirometry outcomes in PD patients have been reported in other cross-sectional studies [2–39, 42–47]. After aquatic exercises, we obtained a mean 16.45% FVC difference from pre-to post-intervention (Assessments 2 and 3).

Immersing the body in the water by the xiphoid process has a 7% to 9% difference in vital capacity in comparison with immersing to the neck [26], with possible negative consequences during immersion [48]. However, carefully planned clinical trials benefit the patients [40], using water resistance to the trunk as inspiratory resistance training. Since ventilation is performed by skeletal striated muscles, it responds to carefully planned intervention programs [49].

Muscle stiffness is one of the PD characteristics, especially in the trunk [5], stiffening the chest and impairing its expansibility [50]. Hence, the proposed exercises are associated with the benefits of heated water to stiffness. Heat transfer and temperature interaction are more intense in immersion [26]. The temperature used in the present study (approximately 33°C) influences muscle tone regulation and diminishes involuntary movements, which are recurrent in neurological cases [26]. Hence, it is believed that less stiff muscles aided more functional chest expansion, leading to higher FVC.

There was no statistically significant difference in FEV1 between the assessments in the present study. FEV1 is closely related to obstructive events and can be influenced by parasympathetic changes in PD. These may be the cause of reported obstructive disorders related to the upper airways [51]. The aquatic intervention program did not impact possible obstructions because it did not have approaches specifically aiming at upper airway obstruction [51].

In fact, FEV1 seems to be little responsive to other modalities of physical activity. Colgrove et al. [14] used yoga physical exercises in interventions, with FVC and FEV1 as outcomes. The yoga intervention lasted 12 weeks, with two sessions a week, which increased FVC after the exercise protocol. On the other hand, as in the present study, they obtained no differences in FEV1. Already the study of Silveira et al. [16] which used two forms of land exercise (functional and aerobic) and assessed chest expansibility, MIP, MEP, FVC, and FEV1–find any FEV1 improvements. The groups only differed in that the functional exercise one had a statistical difference in FVC.

Regarding MVV in the present study, 92.3% of the sample was individually below the recommended in Assessment 1 (MVV >80%). MVV indicates the endurance capacity of the ventilatory system [32]. After the aquatic intervention program, this variable not only significantly increased but also had a moderate effect size (0.596) and the greatest statistical power (0.999).

A cross-sectional study compared PD patients with healthy people and found a statistically significant difference in MVV [52]. On average, those in the PD group had 52.83% of the expected MVV, while the healthy ones had 91.52% of MVV [52]. In another cross-sectional study, Bonjorni et al. [32] demonstrated that MVV correlated in direct proportion with MEP and 6 MWT. These papers show the importance of considering together the respiratory and gait outcomes in PD. Particularly as hypoxic environments increase neurodegeneration, promoting an adequate ventilation volume may prevent neuronal loss [51].

In the present study, the subjective assessment of fatigue was one of the outcomes with the greatest difference before and after the heated pool intervention. There was a 45.18% mean difference in the subjective report of fatigue, with a 0.736 effect size and 0.999 statistical power. Thus, it corroborates the literature, which says that physical activity decreases fatigue and improves motor function and physical capacities in PD [53]. A positive aspect regarding fatigue in the studies on physical activities is the few reports of adverse effects, differently from medication use [54]. Nevertheless, in the present study, fatigue significantly worsened back in Assessment 4, i.e., 3 months after discontinuing the aquatic exercise program, participants reported statistically worse fatigue. Such worsening after finishing the aquatic interventions possibly reflects the progressive nature of PD. These combined results demonstrate firstly that the PD patients' fatigue condition can be changed with aquatic physical exercises and secondly that the stimuli must be continued to maintain the response.

Corroborating these fatigue findings, Ortiz-Rubio et al. addressed land exercises concerning fatigue outcomes in PD patients and the control group. The approach proved to effectively decrease reported fatigue, which was statistically different after the intervention both comparing the groups after the intervention and comparing before and after within the intervention group [55].

It is a complex issue to dissociate subjective fatigue from other findings in PD. Fatigue may be related to respiratory variables, which are much associated with peripheral vasoconstriction in the Metabo reflex mechanism [56]. Metabo reflex can be currently proven in milder land activities [56], reflecting everyday physical-motor difficulties [55]. Muscle stiffness may also be somehow related to the perception of fatigue. In the heated pool intervention, the temperature reduces such excessive tension [57], thus potentially influencing the reported fatigue.

Regarding exercise and gait capacity, PD patients have reportedly reached maximum O_2_uptake and consumption earlier than healthy controls [58], possibly reporting fatigue earlier and with less effort. This shows why PD patients tend to be more sedentary than same-age healthy people [58]. The literature has consolidated reports on the low self-effectiveness of people with PD [59]. Hence, professionals who prescribe physical activities and health administrators must individually identify the barriers to adherence to physical activity [59].

Gait capacity mean values did not reach, in any of the four 6 MWT assessments in the present study, the recommended for healthy older people in the community, which ranges from 392 m to 572 m on average, depending on their age and sex [60]. The body functions involved in the hypotheses that explain poorer 6 MWT performance are the pulmonary, cardiac, cognitive, and orthopedic functions and nutrition [37]. Since gait results from these various functions and structures, as well as other factors, gait intervention must address all these functional capacities. However, the proposed program did not provide significant differences after 3 months of aquatic exercises.

Greater gait distances in PD have been knowingly associated with better-preserved brain mass assessed 9 years later, which was also associated with a significantly lower risk of cognitive impairment [61]. In 6 MWT Assessment 3 (post-intervention), the mean distance was 44.2 m longer than in Assessment 2—which, on average, does not reach the minimum detectable difference for this test in PD, which is 83 m [60].

One hypothesis to explain difficulties in gait capacity is precisely related to ventilatory limitations found in this sample. The neural activity for respiratory muscles and other noble body functions may trigger neuromotor detachment, redirecting energy from peripheral motor activity to essential functions, such as breathing and heartbeat [62]. People with PD possibly have neuromotor detachment as well in situations that require a combined motor and cardiorespiratory responses, as in submaximal tests like 6 MWT. This occurs mainly due to cardiorespiratory complications, with inefficient gaseous exchange incapable of maintaining O_2_ and CO_2_ homeostasis—which commonly occurs in increased dead space, pulmonary hyperinflation, and chronic obstructive pulmonary disease [62].

The aquatic environment potentially provides neuromotor stimuli to trigger gait, with different efferent neuromotor actions from land. Thus, PD patients normally find it easier to walk in the water after adapting to it, due to the change in central pattern [17], which requires greater cortical recruitment—a circuit less dependent on dopamine.

As pointed out by Israel [45], interventions based on motor learning phases aiming at independence in the water enable participants to enjoy an environment with fewer limitations. They can even make movements that would not be possible on land because of the action of forces, especially gravity. Hence, in the therapeutic pool, PD patients explore and activate neuromotor pathways that aid in motor learning and compensation, especially when there are neurofunctional sequelae [40, 45–64].

Nonetheless, besides the slower motor learning rate in PD, they are seemingly dependent on the environment where the skill was trained [65]. This barrier is called set-shifting deficit or stuck-in-set perseveration [66]. A study reported the difficulty of transferring motor skills from the water to the land [67]. The neurophysiological mechanism demonstrating exactly how physical activities can compensate the motor pathways and counterbalance aging and sedentarism in PD is being studied [54].

Lastly, the literature [66] demonstrates that motor neurorehabilitation needs overlap with ventilatory needs in PD. The findings of the present study suggest that aquatic physical therapy stimulates the ventilatory function along with motor therapy in PD. When therapy needs are treated in combination with physical exercises, limitations are prevented or minimized—which is currently an emerging need in PD.

## 5. Conclusion

The aquatic physical exercise intervention program for people with PD positively increased respiratory strength (both inspiratory and expiratory), FVC, MVV, and fatigue. In the control period, no outcome presented differences; however, the respiratory strength, FVC, MVV, and fatigue statistically worsened after the follow-up period (3 months after intervention), receding to levels near those of Assessment 2 (pre-intervention).

## Figures and Tables

**Table 1:** Phase of adjustment.

Exercise	Volume
1st month	
WARM-UP: Gait in circles, holding hands (to the right, left, forward, and backward)	2 min
Bucket handle: Standing; lower limbs apart and partially flexed. Inspiratory exercise combining upper limb abduction/adduction to the water surface; labial frenum prolonged expiration	2 × 5 repetitions, 1 min intervals
Pump lever: Standing; lower limbs apart and partially flexed. Inspiratory exercise combining upper limb flexion/extension; labial frenum prolonged expiration	2 × 5 repetitions, 1 min intervals
Floating with support	2 min

2nd month	
WARM-UP: Gait in circles, holding hands, with pool noodles between lower limbs (to the right, left, forward, and backward)	2 min
Respiratory exercises with short inspirations and prolonged expirations immersed in the water	2 × 5 repetitions, 1 min intervals
Respiratory exercises 2:1 with prolonged expiration immersed in the water	2 × 5 repetitions, 1 min intervals
Floating without support (w/ adaptations, if necessary)	2 min

3rd month	
Warm-up: Gait in circles, not holding hands but maintaining the circle pattern with a ball on the upper limbs and pool noodles between lower limbs; walk to the right, left, forward, in line, and backward	2 min
Respiratory exercises 3:1 with prolonged expiration immersed in the water	2 × 5 repetitions, 1 min intervals
Sliding in the prone position	—
Diving until touching the bottom of the pool	—

**Table 2:** Phase of familiarization with the liquid environment.

Volume (min)	1st month	2nd month	3rd month
4	Transversal rotation	Vertical position floatation	Rolling freely in the water
4	Sagittal rotation	Longitudinal rotation	Mixed/combined rotation

**Table 3:** Phase of specialized therapeutic exercises.

4 min each
*1st month*
Tandem gait forward and backward, holding a small ball
Trunk balance: Sitting on a pool noodle, not touching the feet on the bottom of the floor. Staying still or moving with upper limb movements

*2nd month*
Gait with an obstacle (up and down)
The upper spine: Extending the upper spine from a prone position, holding on to a bar or pool noodle with outstretched upper limbs; associated with respiratory training
The lower spine and gluteal muscles: Taking the lower limbs to the bottom of the pool from a supine position, contracting the abdomen, holding on to a bar with the upper limbs, and having a pool noodle in the lower limbs

*3rd month*
Tandem gait forward and backward, wearing ankle buoyancy cuffs to increase instability
Changing postures: kneeling, partially kneeling, and standing
Ball and bat: In a horizontal (supine or prone) position, embracing the knees (in ball position), then extending the spine and upper and lower limbs (in bat position)
Stretching at the end (2x 30 seconds for each member in each exercise)

*Exercise*
Stretching the ischiotibial and gastrocnemius muscles; one lower limb stretched forward, in unipedal support.
Stretching the quadriceps and iliopsoas muscles; one lower limb with the knee flexed and the hip extended, keeping the ankle behind the body, in unipedal support.
Stretching the quadriceps and iliopsoas muscles; one lower limb with the knee flexed and the hip extended, keeping the ankle behind the body, in unipedal support.
Stretching the large dorsal muscle, standing, hands together over the head, inclining the trunk sideways.
Stretching pectoral muscles; supporting an upper limb against the wall, twisting the trunk to the opposite side of the stretch

**Table 4:** Phase of global organic conditioning.

Exercise (≅12 min of exercise)	1st month	2nd month	3rd month
Stationary bicycle	x	x	x
Jump with upper and lower limb anteroposterior movement	x	x	x
Jumping jacks, taking the upper limbs to the water surface	x		
Swimming with a pool noodle under upper limbs, making front crawl lower limb movements	x		
Standing girdle dissociation, pool noodle under upper limbs, laterally pushing the water surface		x	x
“Swimming”; pool noodle between lower limbs, making displacement movements with upper limbs		x	
Free displacement (swimming), without any floating devices			x

Use the BORG scale every 4 minutes—the professional outside the pool times and takes notes regarding each participant.

**Table 5:** Sample characterization.

Sex (females, males) HY (I, II, III, IV)	5 men, 7 women 1, 4, 3, 4
	Mean ± SD 95% CI (min-max)
Time since diagnosis (years)	8.5 ± 6.58 (3.782012−13.21)
Levodopa dose (mg/day)	570 ± 194.65 (430.75−709.24)
Age (years)	71.3 ± 5.61 (67.28−75.31)
Height (m)	1.61 ± 0.081 (1.55−1.67)
MoCA	21.3 ± 4.66 (17.96−24.63)

Source: the author (2020). Legend: HY: Hoehn & Yahr scale; SD: standard deviation; 95% CI: 95% confidence interval; mg: milligrams; m: meters; kg: kilograms.

**Table 6:** Respiratory variables per assessment period.

Variables	Assessment 1 mean ± SD 95% CI (min-max)	Assessment 2 mean ± SD 95% CI (min-max)	Assessment 3 mean ± SD 95% CI (min-max)	Assessment 4 mean ± SD 95% CI (min-max)	*p* value
MIP (cmH_2_0)	44.1 ± 16.38 (32.38 55.81)	43 ± 15.47^*∗*^ (31.92−54.07)	52.6 ± 19.16^*∗*^^&^ (38.88−66.31)	46 ± 16.12^&^ (34.46−57.53)	*p*=0.001^(GG)^ Effect size = 0.528 Power = 0.961

MEP (cmH_2_0)	37.9 ± 14.31 (27.65−48.14)	36.1 ± 11.72^*∗*^(27.71−44.48)	45.8 ± 12.4^*∗*^^&^ (36.92−54.67)	41.7 ± 10.9^&^ (33.89−49.5)	*p*=0.001 Effect size = 0.567 Power = 0.998

FVC (%)	84.2 ± 19.64 (70.14−98.25)	79.2 ± 18.17^*∗*^(66.2−92.19)	94.8 ± 21.25^*∗*^ (79.59−110)	87.4 ± 17.89 (74.59−100.2)	*p*=0.001 Effect size = 0.374 Power = 0.954

FEV1 (%)	88.1 ± 22.46 (72.02−104.17)	86.09 ± 21.18 (71.85−100.32)	91.5 ± 23.21 (74.89−108.1)	85.8 ± 23.17 (69.21−102.38)	*p*=0.074

FEV1/FVC	81.02 ± 10.04 (73.83−88.20)	82. ± 7.97^*∗*^ (77.18−88.6)	76.53 ± 7.53^*∗*^ (69.14−79.91)	77.1 ± 7.65 (71.62−82.57)	*p*=0.006 Effect size = 0.308 Power = 0.872

MVV (%)	57.2 ± 24.74 (39.5−74.89)	55.95 ± 24.91^*∗*^ (38.12−73.77)	69.8 ± 18.85^*∗*^^&^ (56.31−83.28)	63.1 ± 21.47^&^ (47.74−78.45)	*p* ≤ 0.001 Effect size = 0.588 Power = 0.999

Source: the author (2020). Legend: SD: standard deviation; 95% CI: 95% confidence interval; min: minimum; max: maximum; MIP: maximum inspiratory pressure; MEP: maximum expiratory pressure; cmH_2_O: centimeters of the water column; %: percent; FVC: forced vital capacity; FEV1: forced expiratory volume; FEV1/FVC: tiffeneau index; MVV: maximum voluntary ventilation; ^GG^: sphericity not assumed, greenhouse-Geisser correction used; ^*∗*^: astatistical difference between assessments 2 and 3; ^&^: astatistical difference between assessments 3 and 4.

**Table 7:** Fatigue and 6-minute walk tests per assessment period.

	Assessment 1 mean ± SD 95% CI (min-max)	Assessment 2 mean ± SD 95% CI (min-max)	Assessment 3 mean ± SD 95% CI (min-max)	Assessment 4 mean ± SD 95% CI (min-max)	*p* value
Fatigue scale	4.49 ± 0.93 (3.82−5.16)	4.6 ± 0.58^*∗*^ 4.25−5.09	2.56 ± 0.93^*∗*^ (1.89−3.23)	4.81 ± 0.78^&^ (4.25−5.38)	*p*=0.009 Effect size = 0.736 Power = 0.999

6MWT (m)	400.7 ± 236.15 (231.76−569.63)	388.9 ± 206.08 (241.47−536.32)	433.1 ± 229.23 (269.11−597.08)	372.3 ± 151.54 (263.88−480.71)	*p*=0.144^(GG)^

Source: the author (2020). Legend: 6MWT: 6-minute walk test; SD: standard deviation; 95% CI: 95% confidence interval; ^*∗*^: astatistical difference between assessments 2 and 3; ^&^: astatistical difference between assessments 3 and 4; ^GG^: sphericity not assumed, greenhouse-Geisser correction used.

## Data Availability

Underlying data may be requested from the research authors.
